# Minimally invasive approaches for implantation of left ventricular assist devices

**DOI:** 10.1007/s12055-017-0639-2

**Published:** 2018-02-24

**Authors:** Dominik Wiedemann, Thomas Haberl, Philipp Angleitner, Kamen Dimitrov, Günther Laufer, Daniel Zimpfer

**Affiliations:** Department of Surgery, Division of Cardiac Surgery, Medical University of Vienna, Waehringer Guertel 18-20, 1090 Vienna, Austria

**Keywords:** LVAD implantation, Minimally invasive, Off pump

## Abstract

The era of intracorporal continuous flow pumps has initiated significant success of left ventricular assist device (LVAD) surgery. However, median sternotomy has been the only surgical approach for implantation over many years. During the last decade, less-invasive access ways gained popularity. Within this review, we describe our own clinical experience in minimally invasive ventricular assist device (VAD) surgery and summarize the current scientific literature on this topic.

## Introduction

Till the recent two decades, the success of cardiac surgery was built on the use of cardiopulmonary bypass and full midline sternotomy. Heart surgery is one of the last surgical disciplines implementing less-invasive approaches into the surgical armamentarium. However, today, minimally invasive options for almost every cardiac surgical procedure are available. The variety of access ways includes upper- and lower hemi-sternotomies, lateral mini-thoracotomies, and even thoracoscopic enhanced or fully endoscopic approaches.

Heart transplantation is still the gold standard for the treatment of end-stage heart failure (HF) patients, but lack of donor organs on the one hand and increasing numbers of end-stage HF patients lead to a growing number of left ventricular assist device (LVAD) implantations [1]. In contrast to heart transplantation, left ventricular assist devices are available as “off the shelf product”; therefore, they can be implanted not only as bridge to transplant or in acute settings for patients deteriorating on the waiting list, but also as destination therapy for patients too old for transplantation or with contraindications [2].

The first-generation LVAD devices available on the market were bulky and far too big for any kind of minimally invasive surgical approach. Along with the evolution of these devices and especially the development of continuous flow pumps, the clinical success of ventricular assist device (VAD) surgery started [3].

Together with this development, a significant miniaturization came up, initiating the interest in less-invasive implantation techniques. Despite the fact that median sternotomy still represents the standard approach in LVAD surgery, a variety of different minimally invasive surgical solutions for LVAD implantation is feasible. Depending on the implanted device and on the individual patient’s clinical condition as well as the anatomical situation, it is up to the surgeon to choose not only the right device but also the right surgical approach for the right patient.

The aim of this review is to summarize the current literature on the topic minimally invasive VAD implantation and to explain not only the different surgical approaches with their advantages but also their possible pitfalls, also reflecting the personal experience at our center.

## Methods

A PubMED search was performed between the years 2000 and 2017 with the following keywords: “LVAD implantation,” “minimally invasive VAD,” “less invasive VAD,” “off pump VAD implantation,” and “ventricular assist device.”

The queried articles were then screened for suitability, read, and summarized. The most accurate articles were included in this article. Additionally, this review was enhanced by personal experience and techniques currently used for LVAD implantation at our center. The results are being presented itemized by the implanted device and the surgical approaches possible and in use (Table 1).

**Table 1:** Minimally invasive implantation techniques overview

	Medtronic Heartware HVAD	St. Jude Heartmate II	St. Jude Heartmate III
LVAD only: Surgical access	Bilateral mini-thoracotomy or left thoracotomy + left A. subclavia	Subcostal incision + right thoracotomy or right A. subclavia	Bilateral mini-thoracotomy or left thoracotomy + left A. subclavia
Circulatory support	ECMO or off pump or CPB	CPB	CPB
LVAD + AVR: Surgical access	Left mini thoracotomy + upper hemisternotomy (3rd ICR)	Subcostal incision + upper hemisternotomy (3rd ICR)	Left mini thoracotomy + upper hemisternotomy (3rd ICR)
Circulatory support	CPB	CPB	CPB
+LVAD + TK-repair ± AVR: Surgical access	Left mini-thoracotomy + upper hemisternotomy (4th ICR)	Subcostal incision + upper hemisternotomy (4th ICR)	Left mini-thoracotomy + upper hemisternotomy (4th ICR)
Circulatory support	CPB	CPB	CPB

## Results

### HVAD

The Heartware VAD (HVAD) currently is the smallest CE (Conformité Européenne) marked and FDA (US Food and Drug Administration) approved pump on the market. Therefore, it offers a lot of versatility and possibilities for minimally invasive access surgery. In the scientific literature, the HVAD system is the device with the most minimally invasive implantation experience.

**Fig. 1:**
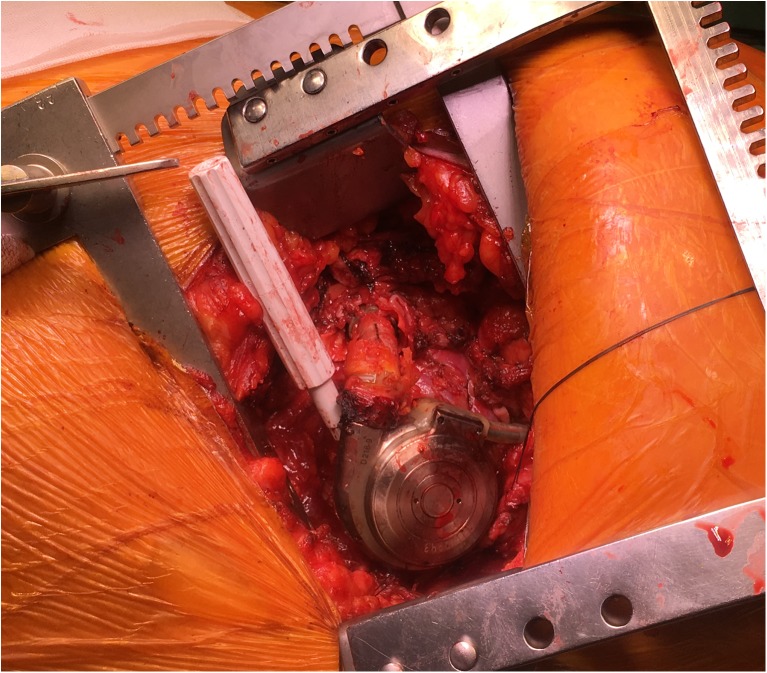
Minimally invasive Heartware HVAD pump exchange

Depending on the surgical requirements and the clinical presentation of the patient, the following minimally invasive approaches are feasible:

For isolated LVAD implantation, access to the left ventricular apex can be gained through a left-sided anterolateral mini-thoracotomy in the fifth intercostal space (Fig. 1); appropriate pump position is verified with TEE guidance, and the HVAD sewing ring is secured to the left ventricular apex with 4–0 prolene in running suture technique.

**Fig. 2:**
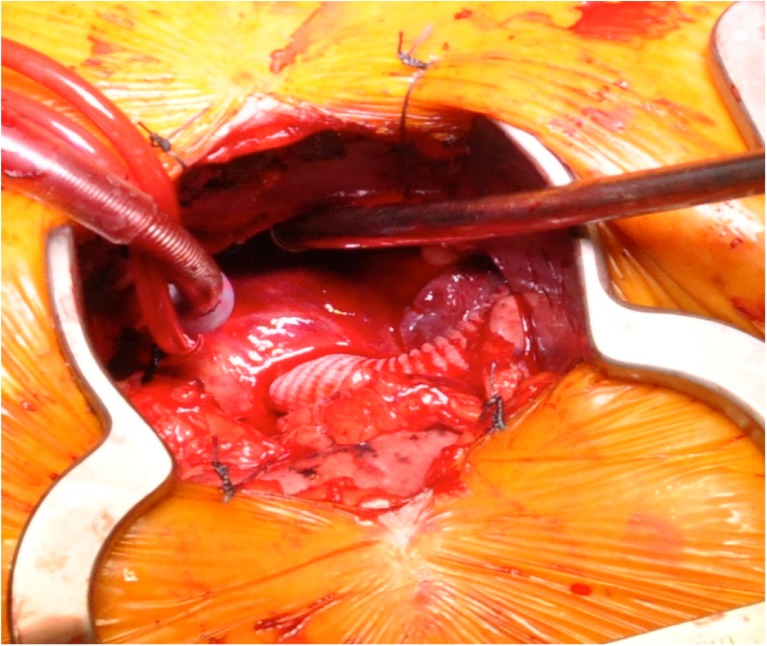
Outflow Graft anastomosis to the ascending aorta

Coring of the left ventricle and connection of the pump can be performed in usual manner similar to open implantation technique. If the outflow graft is to be attached to the ascending aorta, our approach is a right-sided mini-thoracotomy in the second intercostal space. After passing the outflow graft from the left thoracotomy towards the right thoracotomy, a side-biting clamp is attached on the ascending aorta. After adequate incision of the aorta the graft is anastomosed in usual manner (Fig. 2). Finally, the driveline is tunneled into the right upper quadrant of the abdomen.

Our own group published the initial results of the first 27 patients implanted via less-invasive bilateral thoracotomy approach. This series showed rather promising results in a cohort of 85% male patients, 29% Intermacs I, and 22% redo surgeries, and there was a 30-day mortality of 7.5% with no postoperative RV failure, three patients (11%) underwent surgical revision for bleeding, and one (4%) pump thrombosis [4].

Pitfalls:

- Twisting of the outflow graft. Even though we did not experience this complication in our series so far, it is crucial to check the course of the outflow graft from the left towards the right-sided thoracotomy and avoid any kinking or twisting.
- Lung trauma: Gentle handling of the lungs is crucial, in order to avoid postoperative lung injury.
- Tunneling of the driveline: Especially in minimally invasive cases, it is recommended to tunnel the driveline into the right upper quadrant of the abdomen in order to get some distance between the driveline exit-point and the pump/the thoracotomy. Meticulous attention has to be paid not to enter the abdomen or to injury mediastinal structures.

Alternatively to the right-sided thoracotomy, some centers prefer a hemi-sternotomy access for the outflow graft anastomosis. This technique was described by Schmitto et al. in 2012 [5].

Pitfalls:

- Basically, this approach can have the same pitfalls like the bilateral thoracotomy approach.

At our center, we use this approach if additional procedures are necessary: in case of an additional aortic valve replacement, we go for a hemi-sternotomy in the third intercostal space, and in case of an additional tricuspid repair, we aim for the fourth intercostal space.

For anastomosis of the outflow graft, different target vessels beside the ascending aorta can be suitable. One promising target vessel might be the subclavian artery. In case of minimally invasive HVAD implantation, the previously described technique can be used in a similar way. However, there is no need for a second thoracotomy but a left-sided incision two centimeters below the left clavicle is used similar to an incision for a pacemaker implantation. After identification and preparation of the subclavian artery, the outflow graft is tunneled through the second intercostal space. It is recommended to enlarge the interspace and to cover the outflow graft with an additional gore-tex prosthesis at the area passing the ribcage in order to avoid kinking of the graft.

Before anastomosing the outflow graft, a suitable position on the inferior side of the subclavian artery is identified. After performing the anastomosis and establishment of the LVAD flow, a banding distal to the anastomosis is attached around the subclavian artery aiming for equal blood pressure levels in both arms [6].

The subclavian approach seems very appealing in cases of severe calcification of the ascending aorta; also, VAD implantation in redo scenarios can be facilitated by so avoiding re-sternotomy. However, there are concerns regarding hyperperfusion of the arm and issues related to a mismatch between the diameter of the outflow graft and the size of the target vessel which might be predisposing for pump thrombosis.

Pitfalls:

- Hyperperfusion of the arm: As already mentioned, banding of the subclavian artery can at least reduce possible hyperperfusion of the arm.
- Size mismatch: Preoperative computed tomography (CT) scan is crucial especially for this kind of approach. If the left subclavian artery is extraordinary small, this approach should be avoided. In general, it is recommended to make a relatively large anastomosis to establish optimal flow.
- Preoperative implanted ICD (implantable cardioverter defibrillator): Even though the ICD device might be in your way, it is still possible to use this approach. Attention has to be paid not to injury the ICD leads, and it is recommended to re-adapt some tissue between the ICD and the anastomosis in order to form a barrier for possible infections.

Apart from the subclavian artery, the outflow graft can also be attached to the descending aorta. Interestingly, the first LVAD implantation in 1963 by Crawford and Liotta was performed through thoracotomy with the outflow graft anastomosed towards the descending aorta. So actually already, the first VAD was a less-invasive implantation. The only major difference to contemporary implantation techniques was that the inflow of the VAD was not inserted into the left ventricular apex; instead, it was introduced into the left atrium [7, 8].

Pitfalls:

Although the descending aorta is still used as a target for the outflow graft today, it has never reached the level of a standard technique due to technical problems and postoperative issues like thrombus formation in the ascending aorta. Nevertheless, this approach can still represent a bail out scenario in cases where other vessels are not suitable due to calcifications or insufficient diameters.

### Heartmate II

The Heartmate II is the device with the longest clinical experience of all currently used continuous flow pumps, but it is also the largest of the contemporary LVAD devices [9].

**Fig. 3:**
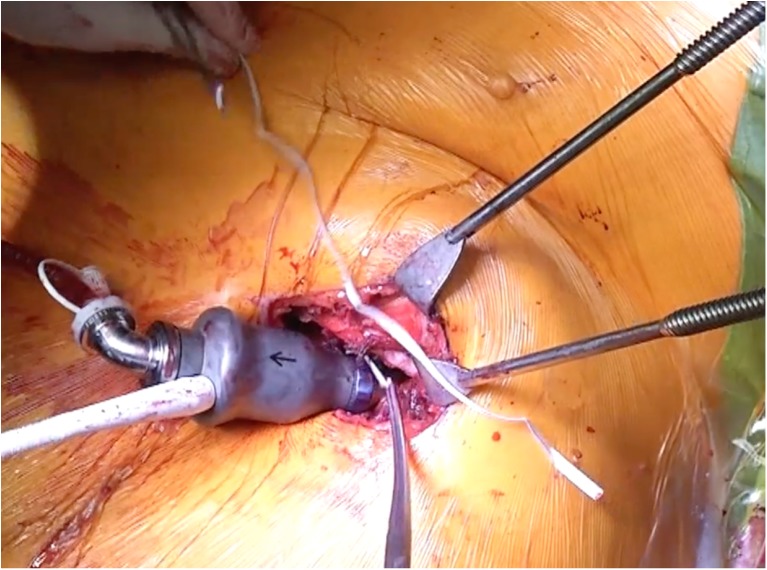
Thoratec HM II insertion through subcostal incision

Nevertheless, minimally invasive implantation of this pump is also feasible; however, the surgical approach has to be modified slightly. Instead of a left-sided thoracotomy a subcostal incision is used running along the left-sided ribcage (Fig. 3). After division of the abdominal muscles, preparation anteriorly to the diaphragm is performed. Through this access, the pericardium can be divided and the left ventricular apex easily identified. We are proponents of running suture technique also when securing HM II inflow cannula towards the left ventricle; however, also single stitches can be used.

**Fig. 4:**
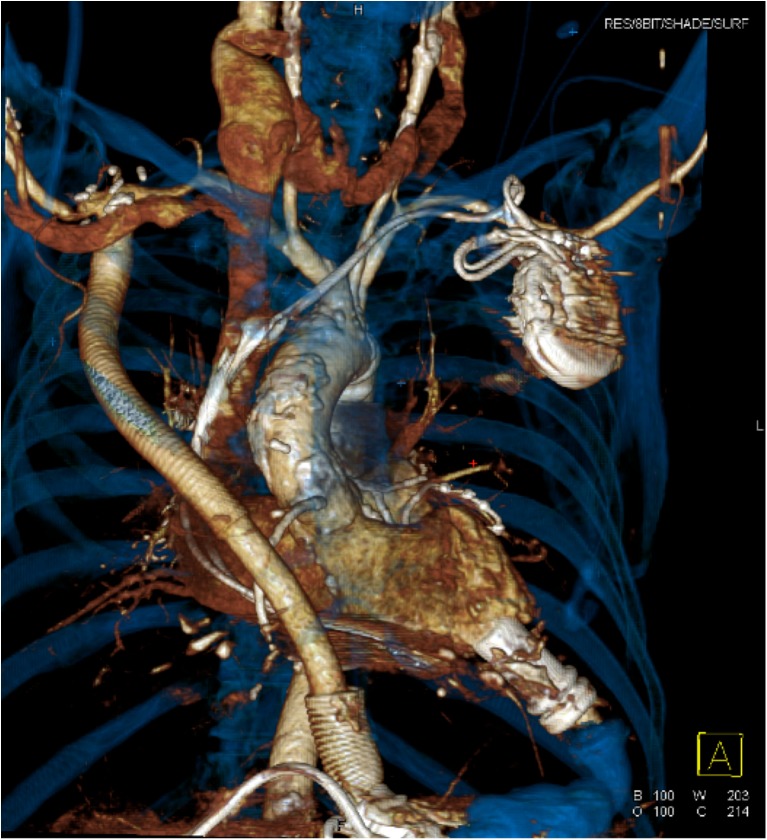
CT scan reconstruction of the outflow graft to the right subclavian artery

Aiming for the subclavian artery as the target vessel for outflow graft anastomosis is also feasible in HMII; however, due to the design of the device, you have to use the right-sided subclavian artery instead of the left one like in HVAD cases (Fig. 4). Exchange of the Heartmate II in case of pump thrombosis can also be performed through this access if needed [10]. Levin AP et al. described a series of 232 Heartmate II patients of which 28 required 36 pump exchanges. They found 100% survival in their subcostal (minimally invasive) exchanges whereas only 63% survived the exchange when performed via sternotomy approach while both groups had the same (high) risk of thrombus recurrence (31%).

Pitfalls:

- Due to the relatively large size of the HMII pump, it is necessary to disconnect the modular portions of the pump in order to perform the implantation properly via this approach.
- Entering of the abdomen should be avoided.

### Heartmate III

Heartmate III is the latest CE marked continuous flow LVAD device on the market. It is especially appealing due to promising low rates of pump thrombosis in the CE mark trial [11, 12].

While within the CE mark trial, the 30-day survival rate was excellent with 98% and the rate of adverse events (bleeding 30%, arrhythmia 28%, infections 20%, strokes 4%) was throughout comparable to other contemporary devices. Especially, the 0% pump-thrombosis rate appears promising [11].

**Fig. 5:**
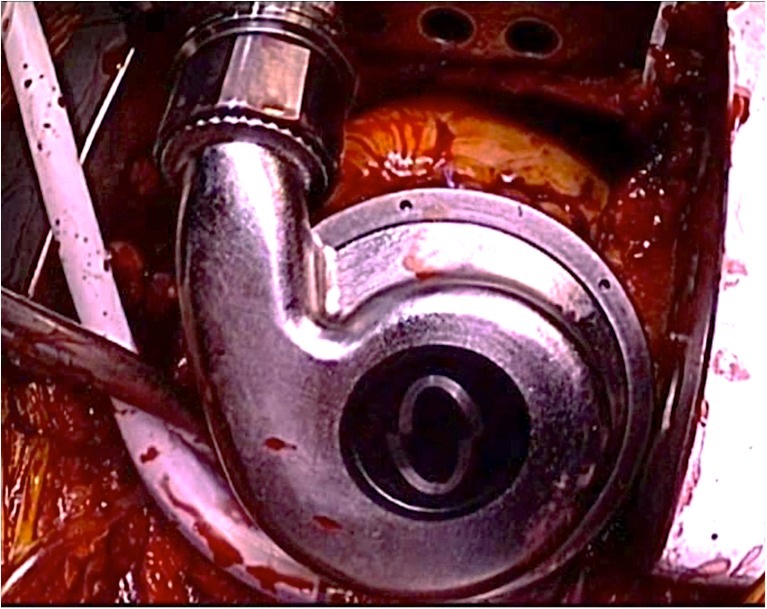
View of thoratec HM III through left-sided thoracotomy

The profile of the pump is a little higher than the profile of the HVAD and the sewing ring is different. However, in minimally invasive implantation, techniques similar to the described HVAD implantation can be used (Fig. 5).

To implant the pump through a left-sided thoracotomy, a slightly greater incision as for the HVAD implantation is recommended; however, there are also special instruments under development facilitating the implantation of this pump through this access. Due to the different designs of the sewing ring, we recommend 2–0 prolene in a running suture technique. For anastomosis of the outflow graft, the previously described techniques and approaches like in less-invasive HVAD implantation can be used for this in same fashion.

Pitfalls

- Apart from the same considerations like in the minimally invasive HVAD implantations, it has to be considered that the pump has a higher profile and a larger sewing ring; therefore, a slightly larger thoracotomy is recommended.

### On pump-off pump or implantation on ECMO (extracorporeal membrane oxygenation)?

In terms of minimally invasive surgery, not only the localization and the size of the incision are a matter of debate, but also the use of cardiopulmonary bypass.

Most of the implantations at our department are still being performed on full cardiopulmonary bypass support. In case of minimally invasive VAD implantation, we either cannulate the ascending aorta and the right atrium directly over the right-sided thoracotomy or hemi-sternotomy; alternatively, we perform cannulation of femoral vessel or cannulation of the subclavian artery.

When aiming for an off-pump approach we use the following strategies:

After attachment of the sewing ring, temporary pacing-wires are placed for rapid pacing. Then, catecholamine dosage is increased and after initiation of rapid pacing, we wait a few seconds until the heart stops ejecting. Then, the left ventricle is incised and the coring device is introduced. After that, rapid pacing is stopped and the patient stabilized again.

After a few minutes of stabilization, a second period of rapid pacing is induced again, coring of the left ventricle is finalized and the pump is introduced quickly.

We use the same implantation strategy also in cases of implantation on ECMO. Whether off pump implantation or implant on ECMO has advantages or disadvantages in comparison to full cardiopulmonary bypass (CPB) usage in that patients is not proven yet. Clinical trials would be warranted.

In the literature, there is only one single series with 26 patients receiving an HVAD in less-invasive off pump technique, which was published by Strueber et al. in 2014. In this series 1, conversion to CPB was needed. All patients survived 90 days, and the authors concluded that this approach is safe and might result in favorable outcome [13].

Pitfalls:

- Especially not only in off pump cases but also in ECMO cases, connection of the pump should be performed quickly in order to avoid blood loss and hemodynamic instability.
- In off pump cases, it can be reasonable to place guiding wires in the groin for emergency cannulation if needed.
- Prior to the coring process, it is recommended to load the patient with vasopressive agents to minimize the hemodynamic trauma during the off pump implantation process.

## Discussion

Like in other cardiac surgical procedures, minimally invasive approaches are becoming more commonly used, also in the field of VAD implantation. It remains a matter of debate if these approaches offer advantages in comparison to standard full-sternotomy access. Possible advantages may include the following: reduction of surgical trauma, better preservation of right ventricular function, less bleedings, shorter mechanical ventilation time, shorter ICU stays, less adhesions at time of transplantation, less infection, and improved survival.

In our own center, we see a trend towards reduced requirement of inotropic support and blood products in minimally invasive access surgery. An undeniable advantage of the minimally invasive implantation technique is the lack of adhesions at time of transplant, which tremendously facilitates heart transplantation and also reduces ischemia time resulting in improved graft performance. Rojas et al. published a prospective analysis comparing destination therapy patients implanted less-invasive (*n* = 20) with a group of median sternotomy implants (*n* = 26). They found a lower incidence of bleeding, reduced need for inotropic support, and even a tendency towards lower mortality in the less-invasive group [[14].]

Whether all the positive expectations towards minimally invasive VAD surgery will become true and moreover will result in improved clinical outcome still has to be proven. To summarize, LVAD surgery has undergone an evolution and reflects nowadays a compendium of different approaches for various clinical situations. For the LVAD surgeon of the twenty-first century, it is of outmost importance to establish individual strategies for each implantation.
